# Unroofed coronary sinus newly diagnosed in adult patients after corrected congenital heart disease

**DOI:** 10.1007/s12471-014-0533-0

**Published:** 2014-03-04

**Authors:** A. J. Pérez Matos, R. N. Planken, B. J. Bouma, M. Groenink, A. P. C. M. Backx, R. J. de Winter, D. R. Koolbergen, B. J. M. Mulder, S. M. Boekholdt

**Affiliations:** 1 Department of Cardiology, Academic Medical Center, PO Box 22660, 1100 DD Amsterdam, the Netherlands; 2 Department of Cardiology, St. Antonius Hospital, Koekoekslaan 1, 3435 CM Nieuwegein, the Netherlands; 3 Department of Radiology, Academic Medical Center, PO Box 22660, 1100 DD Amsterdam, the Netherlands; 4 Department of Pediatric Cardiac Surgery, Academic Medical Center, PO Box 22660, 1100 DD Amsterdam, the Netherlands

**Keywords:** Unroofed coronary sinus, Congenital heart disease, Noninvasive imaging, Atrial septal defect

## Abstract

Patients with congenital heart disease corrected in early childhood may later in life present with cardiac symptoms caused by other associated congenital anomalies that were initially not diagnosed. Nowadays, several noninvasive imaging modalities are available for the visualisation of cardiac anatomy in great detail. We describe two patients with an unroofed coronary sinus, a rare congenital anomaly which could be diagnosed using a combination of modalities including echocardiography, cardiac CT and cardiac MRI.

## Background

**Fig. 1:**
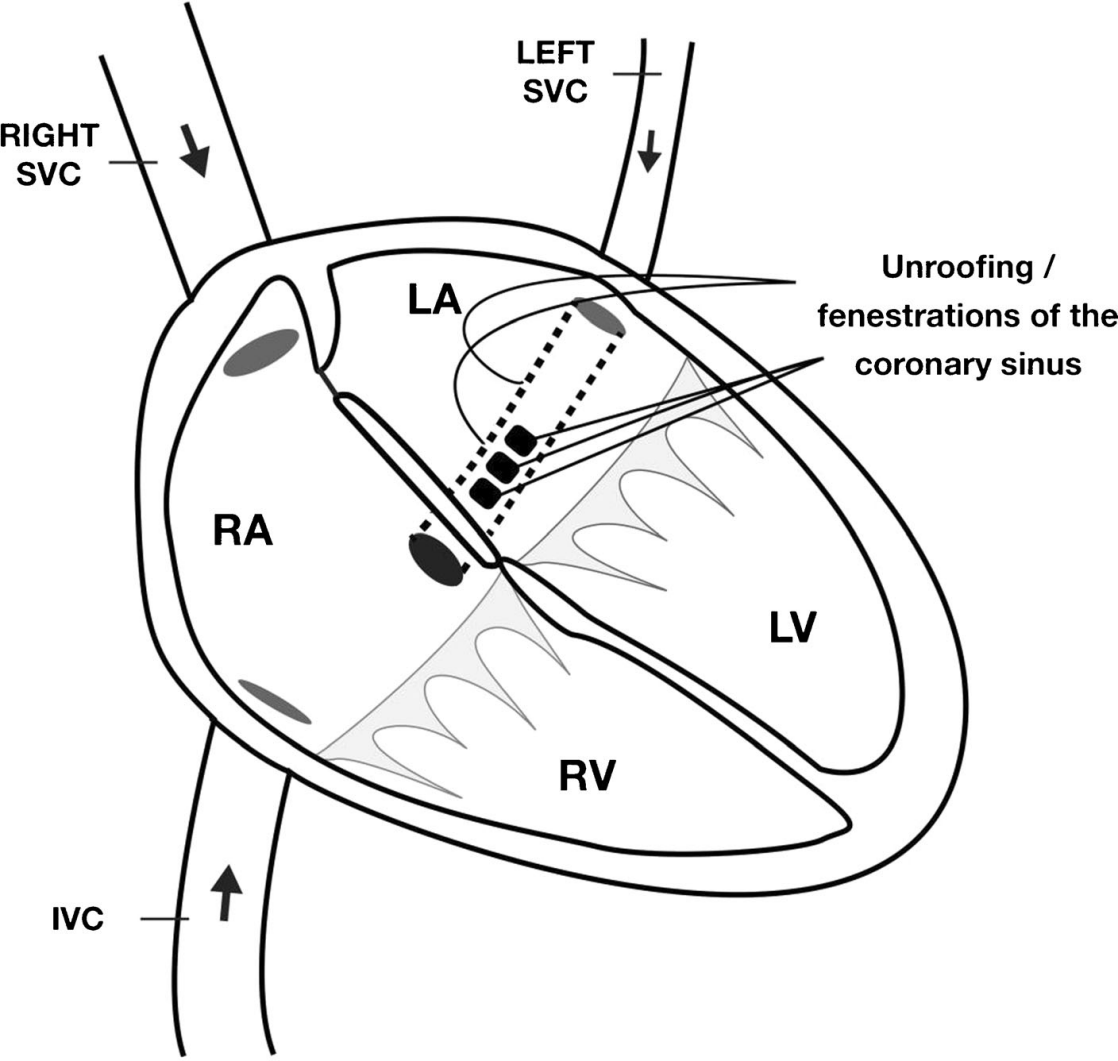
Schematic drawing of an unroofed coronary sinus. *SVC*  superior vena cava, *IVC* inferior vena cava, *LA* left atrium, *RA* right atrium, *RV* right ventricle, *LV* left ventricle

An unroofed coronary sinus (UCS) is a rare congenital heart anomaly, first described in 1965 by Raghib et al. [1] An UCS is often associated with a persistent left vena cava superior (PLVCS) and other congenital heart defects including ventricular septum defect, atrioventricular septal defect, cor triatum and tetralogy of Fallot [1–3]. Kirklin and Barratt-Boyes classified the anatomical variants of UCS as follows: type I: completely unroofed coronary sinus with a PLVCS; type II: completely unroofed coronary sinus without PLVCS; type III: partially unroofed coronary sinus in the mid-portion; and type IV: partially unroofed coronary sinus in the terminal portion [4]. Under normal circumstances, the coronary sinus drains blood from the cardiac veins into the right atrium. An UCS is caused by partial or complete absence of the partition between the coronary sinus and the left atrium, which leads to communication between the left and right atrium and interatrial shunt. In addition, the communication between the coronary sinus and the left atrium causes aberrant drainage of the cardiac venous system into the left atrium (see Fig. 1 for a schematic drawing). If an associated PLVCS exists, the systemic venous return via the PLVCS is also drained aberrantly into the left atrium. As a result of the right-to-left communication, there is mild desaturation as well as an increased risk of cerebral emboli and brain abscess [1]. On the other hand, the left-to-right shunt causes volume overload of the right ventricle which can cause right-sided heart failure.

## Case A

A 24-year-old male visited our outpatient clinic for follow-up after totally corrected tetralogy of Fallot at the age of 1 year. At the age of 16 he had severe pulmonary regurgitation with volume overload of the right ventricle and ventricular arrhythmia, for which he underwent pulmonary valve replacement (PVR) with a homograft. He had remained under annual surveillance at the Department of Paediatric Cardiology until the age of 18, but upon transfer to the Department of Cardiology he withdrew from surveillance for several years. When he came back at the age of 24, he was asymptomatic and had a good exercise capacity. On auscultation he had a loud left parasternal systolic murmur at the second intercostal space. His saturation was 98 %. No other abnormalities were noticed. The electrocardiogram showed sinus rhythm with right axis deviation and a right bundle branch block.

**Fig. 2:**
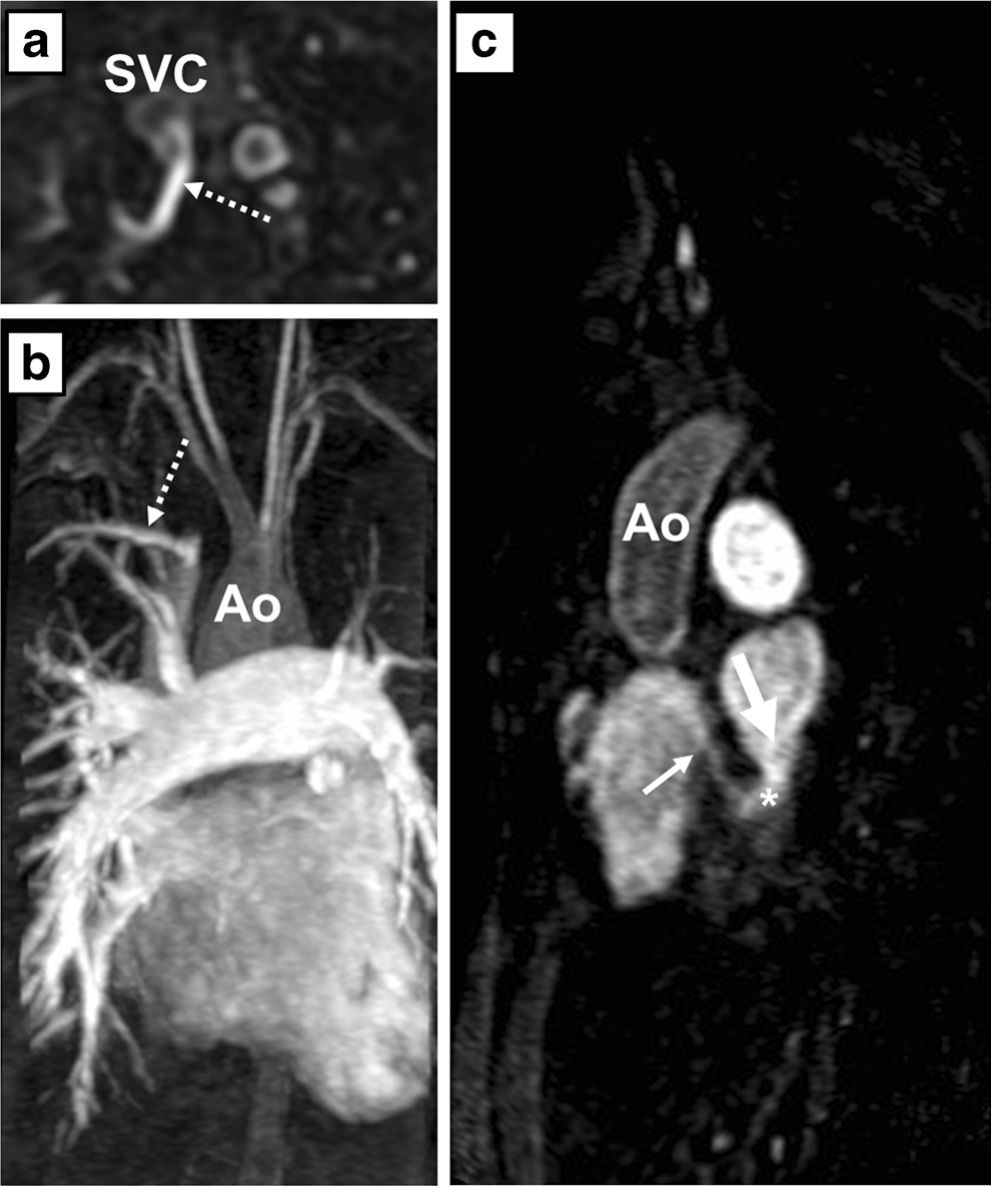
Pulmonary angiogram by contrast-enhanced magnetic resonance angiography (MRA) of patient A. Axial (**a**) and sagittal (**c**) oblique multiplanar reconstruction and coronal maximum intensity projection (**b**) of the unroofed coronary sinus (*), the coronary sinus defect (*thick arrow*) and the entry of the coronary sinus into the right atrium (*thin arrow*). The right upper lobe drains into the right superior vena cava (*dotted arrow*). *RA* right atrium, *LA* left atrium, *Ao* aorta, *SVC* superior vena cava

**Fig. 3:**
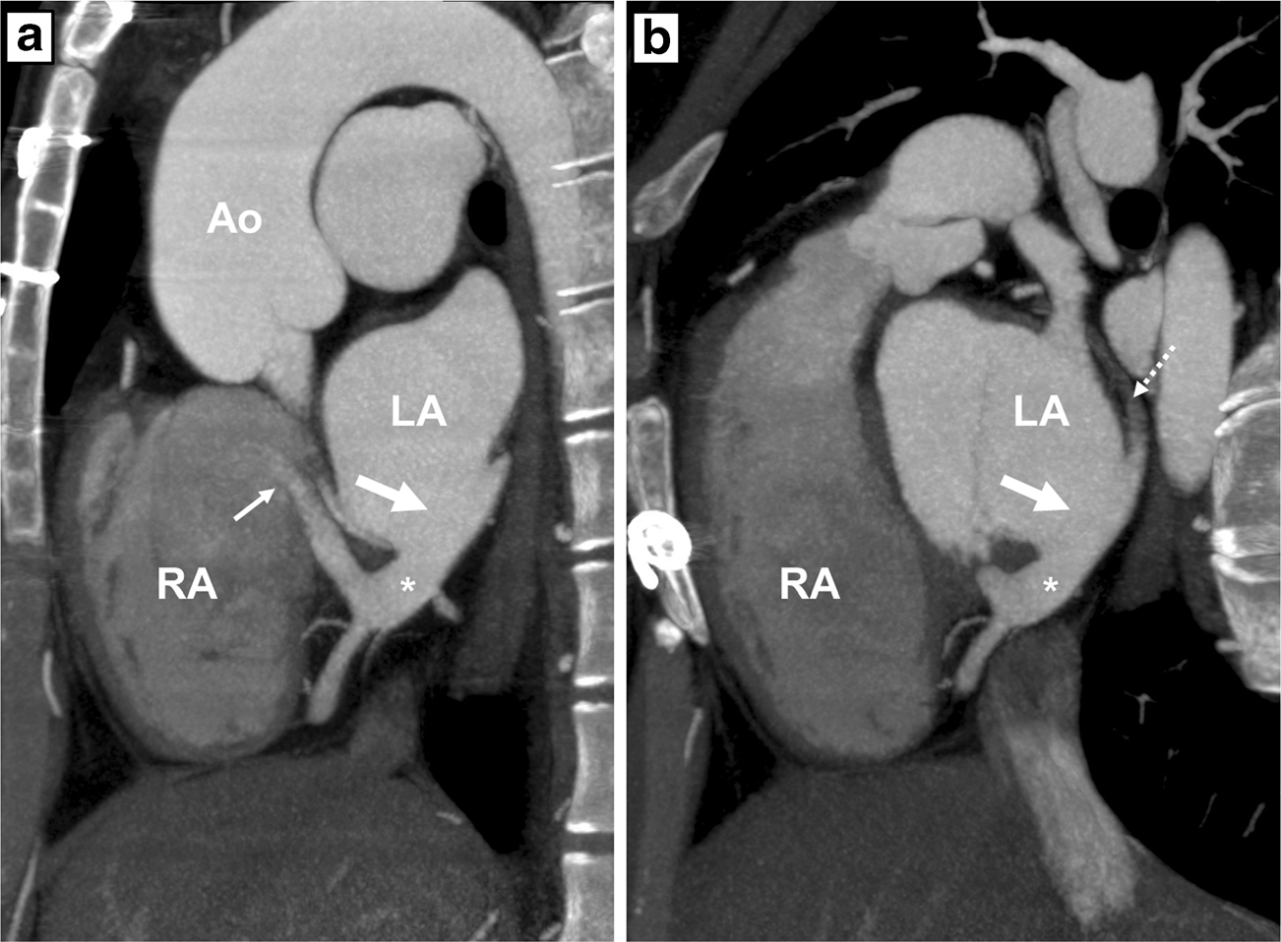
Computed tomography angiography (CTA) images after intravenous contrast administration in patient A. **a**: Sagittal oblique multiplanar reconstruction of the unroofed coronary sinus (*), the coronary sinus defect (*thick arrow*) and the entry of the coronary sinus into the right atrium (*thin arrow*). **b**: Note that the great cardiac vein is not opacified by intravenous contrast (*dotted arrow*) and that the coronary sinus shows opacification comparable to the left atrium. *RA* right atrium, *LA* left atrium, *Ao* aorta

Transthoracic echocardiography showed a dilated and hypertrophied right ventricle with a moderately decreased systolic function and a moderate pulmonary valve stenosis (peak gradient 42 mmHg) with a mild pulmonary regurgitation. The pulmonary arterial pressure (PAP) could not be estimated. He underwent MRI to quantify the extent of right ventricular (RV) dilatation and dysfunction. The right ventricle was moderately dilated (RV end-diastolic volume 304 ml versus left ventricular end-diastolic volume 198 ml) and systolic function was mildly impaired (ejection fraction 45 %). Flow through the pulmonary artery was larger than through the aorta, and there was a consistently larger stroke volume of the right compared with the left ventricle, which could not be explained by valvular regurgitation. Magnetic resonance angiography revealed a partial aberrant pulmonary venous connection (PAPVC) of the right upper lobe to the superior caval vein (Fig. 2). Subsequently, a cardiac CT was performed, which revealed an UCS type II (Fig. 3). In addition, a pseudo-aneurysm was seen at the distal anastomosis of the homograft.

## Case B

A 56-year-old woman was referred to our outpatient clinic because of progressive exertional dyspnoea. She had undergone closure of a ventricular septum defect when she was 3 years old. At the age of 49, she developed paroxysmal atrial fibrillation. Because she was highly symptomatic and the atrial fibrillation turned out to be resistant to medical therapy, she underwent His bundle ablation and VVI-pacemaker implantation. There were bilateral superior caval veins but no connection between the left subclavian vein and the right superior caval vein, so the pacemaker was implanted on the right side. She had remained asymptomatic for 5 years. She was now referred to our outpatient clinic because of deterioration of her exercise capacity and progressive dyspnoea on exertion.

**Fig. 4:**
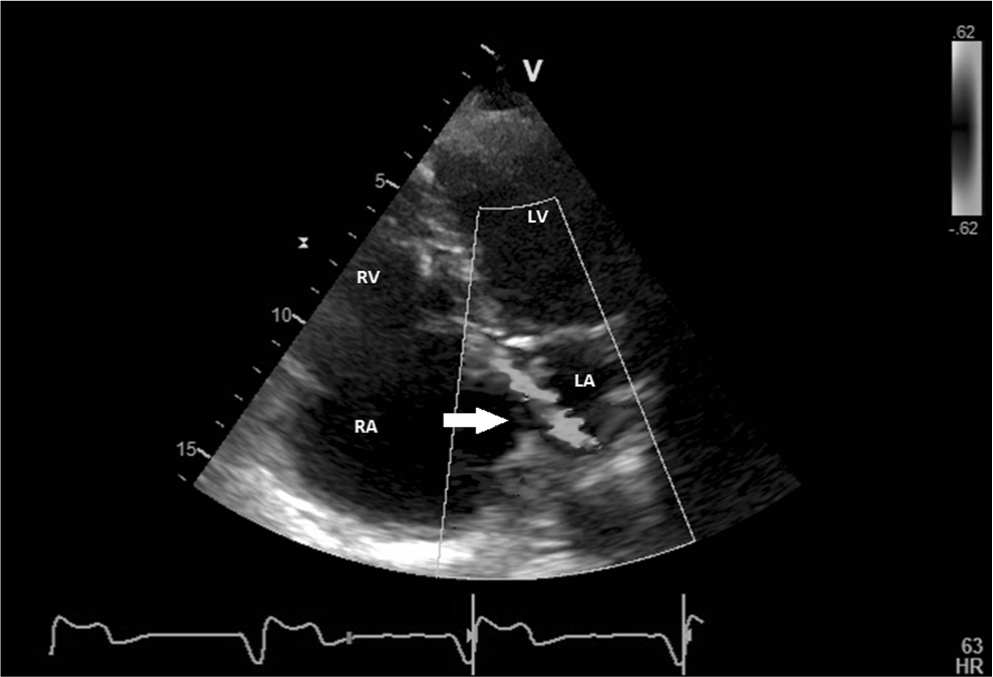
Transthoracic echocardiography, four-chamber view of patient B showing an abnormal flow signal from the roof of the left atrium to the interatrial septum on colour Doppler (*arrow*). *RA* right atrium, *RV* right ventricle, *LA* left atrium and *LV* left ventricle

**Fig. 5:**
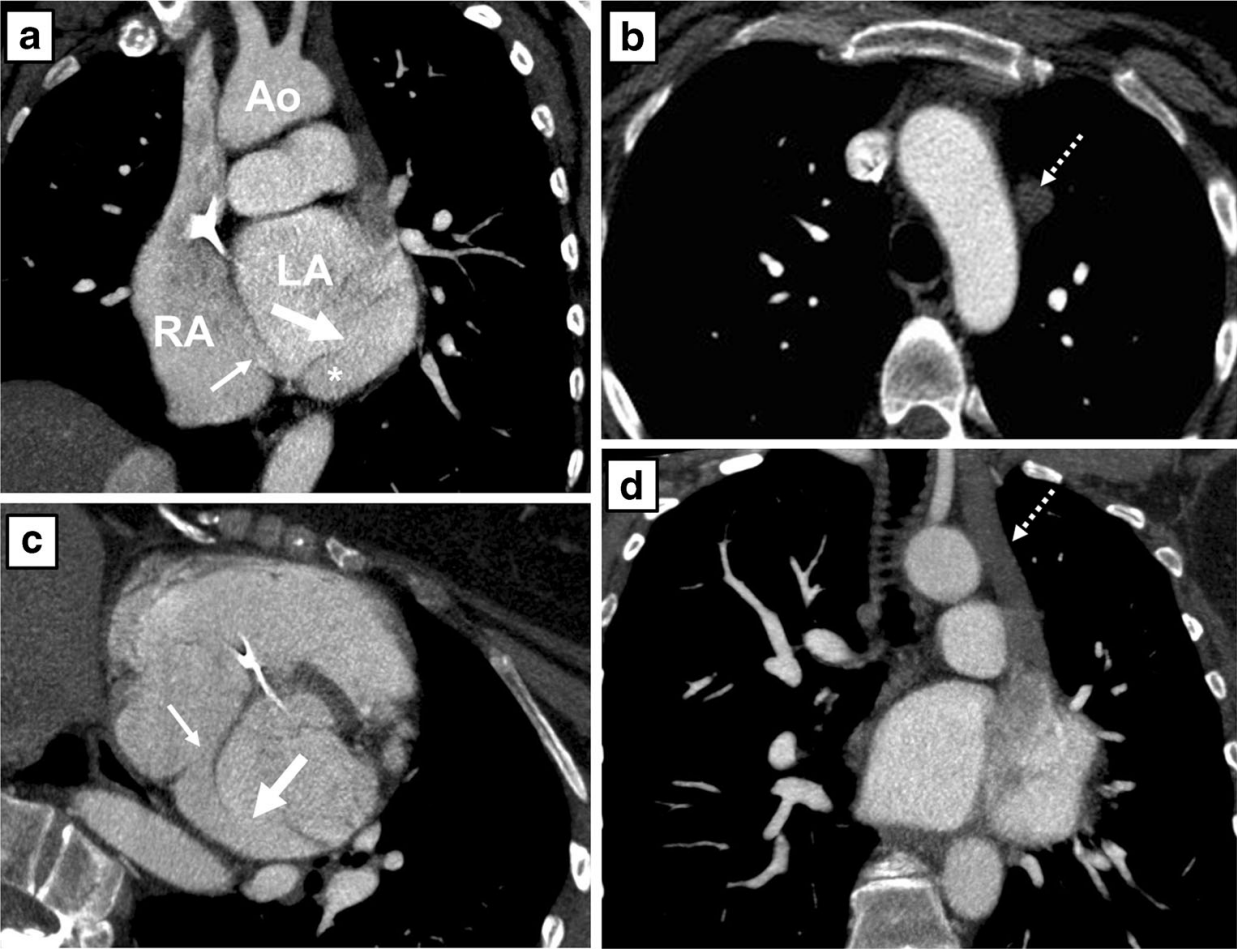
Computed tomography angiography (CTA) images after intravenous contrast administration of patient B. Coronal (**a&d**) and axial (**b&c**) oblique multiplanar reconstructions of the unroofed coronary sinus (*), the coronary sinus defect (*thick arrow*) and the entry of the coronary sinus into the right atrium (*thin arrow*). Note the persistent left superior vena cava (**c&d**). *RA* right atrium, *LA* left atrium, *Ao* aorta

At physical examination, her saturation was 92 % at rest, which dropped to 72 % after mild exercise. Transthoracic echocardiography revealed a moderately dilated right ventricle with a normal systolic function and severe tricuspid valve insufficiency due to tricuspid annular dilation in combination with retraction of the septal leaflet caused by the pacemaker lead. The systolic PAP was estimated at 30 mmHg using the tricuspid insufficiency signal. Colour Doppler showed a flow signal from the left to the right atrium suggesting an atrial septum defect (ASD) (Fig. 4). After infusion of agitated saline contrast, a right-to-left shunt at atrial level was seen, suggesting a patent foramen ovale or an ASD with right-to-left shunting. Transoesophageal echocardiography confirmed an 8 mm defect in the posterior part of the interatrial septum with bidirectional flow matching the diagnosis of ASD type UCS. Cardiac CT showed a PLVCS draining into a dilated coronary sinus, which was partially unroofed causing communication with the left atrium (Fig. 5).

## Discussion

These two patients illustrate different clinical presentations of an UCS as a result of differences between the size of the defect and the degree of left-to-right shunt. Symptoms associated with UCS may vary from none at all, to nonspecific complaints of exertional dyspnoea, to right-sided heart failure due to chronic volume overload as in case B [5, 6]. Snijder et al. described a patient very similar to case B who suffered anginal complaints [7].

These two patients were previously diagnosed with congenital heart disease which had been corrected in childhood. Still the UCS was not diagnosed at that time, demonstrating the difficulty of the diagnosis in a time when imaging modalities were not so advanced as they are nowadays. Current imaging modalities offer the possibility to image the 3D anatomy of the entire heart, which can help in the diagnosis of rare anomalies such as UCS.

In both cases, the diagnostic process was challenging due to concomitant abnormalities. For instance, if the RV dilatation in case A had been considered a consequence of the history of pulmonary regurgitation, for which he had undergone PVR in 2004, then additional diagnostic work-up could have been deemed unnecessary. Similarly, if the PAPVC of the right upper lobe had been considered the cause of RV dilatation despite its small size, then it is unlikely that the UCS would have been discovered during additional tests. It should also be considered that in case A, the size of the left-right shunt was probably limited as a consequence of elevated RV systolic pressure (RVSP) due to progressive pulmonary stenosis. In case B, if the RV dilatation and dysfunction had been ascribed to chronic pacing and tricuspid regurgitation due to the pacemaker lead, then additional diagnostic work-up would not have been performed.

At present, none of the four diagnoses in patient A, i.e. UCS type II, PAPVC of the right upper lobe, moderate pulmonary valve stenosis and a pseudo-aneurysm of the distal anastomosis of the homograft form an indication for intervention. MRI flow measurements across the aorta and pulmonary artery showed that the shunt caused by the UCS and the PAPVC is mild as a consequence of the increased RVSP due to the pulmonary stenosis. The fact that the left-to-right shunt at atrial level is limited is also supported by the fact that the terminal portion of the coronary sinus between left and right atrium was not dilated. The pseudo-aneurysm at the distal anastomosis of the homograft is also not an indication for operation in itself since the risk of progression or rupture is small in the low pressure pulmonary circulation. We decided to postpone surgery until the progressive pulmonary stenosis becomes severe enough to justify surgery. According to the ESC guidelines for the management of grown-up congenital heart disease, an operation is indicated for pulmonary valve stenosis when the peak gradient is >64 mmHg or the RV pressure exceeds 60 mmHg in symptomatic patients (class IC). In asymptomatic patients it should be considered when a severe pulmonary valve stenosis is accompanied by other criteria such as progressive RV dilation or RV systolic dysfunction [8]. In general, reoperation in this population of patients with congenital heart disease is not without risk. Zomer et al. demonstrated that the long-term survival for patients with reoperations in adulthood is inversely correlated to the number of surgeries [9].

The symptoms of patient B are caused by the UCS, which has a pathophysiology similar to an ASD, namely a left-to-right shunt resulting in a volume overload for the right ventricle and the risk of developing pulmonary arterial hypertension. In addition, the PLVCS drains aberrantly in the left atrium causing desaturation. Her symptomatology is most likely explained by progressive pulmonary arterial hypertension, which also causes desaturation due to right-left shunting through the coronary sinus during exercise. The treatment of choice is to close this defect if there is no Eisenmenger syndrome. She was treated with bosentan for 3 months and subsequently underwent right heart catheterisation. The pulmonary pressure measured at 40/17 (mean 24) mmHg was well below 2/3 of the systemic arterial pressure of 114/61 (mean 82) mmHg. The Qp:Qs was 1.4 to 1.0, including the right-to-left shunt. The decision was made for surgical repair of the UCS. While inspecting the left atrium, the PLVCS was confirmed to drain just posteriorly of the left atrial appendage. The coronary sinus was largely unroofed, with the exception of the most distal 2.5 cm, which formed a tunnel connecting the left and right atrium. The coronary sinus was reconstructed with a xenopericard patch over a length of 11 cm. The pacemaker lead was removed from the tricuspid septal leaflet and sutured at the anteroseptal commissure. A tricuspid annuloplasty ring (32 mm) was placed to reduce the annular dimension. At the four-month postoperative follow-up she reported marked improvement in her exercise capacity.

The coronary sinus can be visualised by transthoracic echocardiogram, although this can be difficult because it is located deep posteriorly in the left atrioventricular groove. As with the pulmonary veins, when the coronary sinus is draining into the posterior wall of the left atrium this is not well delineated by transthoracic echocardiogram [10]. In the parasternal long-axis view, it is easy to visualise the coronary sinus when it is dilated. However, demonstrating a defect by echocardiography can be difficult because of the limited sonic window. Transoesophageal echocardiogram is more accurate but it still has the limitations as mentioned above. A dilated coronary sinus should raise the suspicion of a UCS, but in patients with a dilated coronary sinus due to PLVCS, the diameter of the coronary sinus itself does not help to differentiate between those with and without UCS. Kim et al. found that in patients with UCS, the mean diameter of the coronary sinus standardised to the patient’s body surface area was 15 ±4 mm/m^2^, which is similar to that of patients with a PLVCS without UCS (15 ± 6 mm/m^2^) and significantly greater than of patients with normal anatomy (7 ± 2 mm/m^2^) [11].

Cardiac CT and MRI are useful for the detection of UCS and other associated anomalies [10, 12]. Cardiac CT has a higher spatial resolution than MRI and in addition the capability of multiplanar reconstruction. Therefore, it will result in better anatomic information [10]. It is also useful for imaging of uncooperative patients because of the short scanning time. An important disadvantage of CT is the radiation exposure, although with modern scanners the radiation dose is quite low. MRI is also very useful for the visualisation of deep lying structures, and does not require radiation [10]. In addition, MRI can provide functional information about left and right ventricular volumes and function, as well as valvular function and shunt size. In our opinion, CT and MRI are complementary in the diagnosis of rare congenital cardiac anomalies such as UCS. In general, these imaging modalities should be used in the context of a targeted diagnostic process, as exemplified by these two cases with incompletely explained RV dilation and dysfunction.

For patients awaiting surgical correction or percutaneous interventions for congenital heart disease a comprehensive imaging work-up should be considered to better appreciate the extent of disease. Technical developments over the last decade have resulted in a substantial increase in image quality that results in a better appreciation of anatomy. Furthermore, the decrease in radiation dose and contrast use make computed tomography imaging more accessible.

Treatment of a UCS consists of surgical rerouting of the coronary sinus and possibly the associated PLVCS to the right atrium using pericardium, a xenopericard patch or left atrium wall. Some defects are so small that closing the holes in the roof of the coronary sinus is sufficient. In the case of an associated PLVCS, when a communicating brachiocephalic vein is present, then the PLVCS can be ligated [12]. However, when the PLVCS ends in the left atrium the brachiocephalic venous bridge is usually absent.

In summary, the diagnosis of a UCS should be considered in a patient with an unexplained enlarged right ventricle, hypoxaemia or desaturation. Clinical suspicion should be particularly high in patients with known congenital heart disease, because there is a strong association between UCS and other anomalies. This diagnosis can remain undetected for decades even in patients who have already undergone cardiac surgery. However, clinical suspicion in combination with modern imaging techniques can help to establish this rare diagnosis.
